# The Effect of Exercise on the Older Adult's Blood Pressure Suffering Hypertension: Systematic Review and Meta-Analysis on Clinical Trial Studies

**DOI:** 10.1155/2020/2786120

**Published:** 2020-09-15

**Authors:** Mohsen Kazeminia, Alireza Daneshkhah, Rostam Jalali, Aliakbar Vaisi-Raygani, Nader Salari, Masoud Mohammadi

**Affiliations:** ^1^Department of Nursing, School of Nursing and Midwifery, Kermanshah University of Medical Sciences, Kermanshah, Iran; ^2^School of Computing, Electronics and Maths, Coventry University, London, UK; ^3^Department of Biostatistics, School of Health, Kermanshah University of Medical Sciences, Kermanshah, Iran

## Abstract

**Background:**

Senescence refers to spontaneous and progressive irreversible degenerative changes in which both the physical and psychological power diminish significantly. Hypertension is the most common cardiovascular disease in the elderly. Several studies have been conducted regarding the effect of exercise on reducing the blood pressure of the elderly, which have found contradictory results. One of the uses of meta-analysis study is responding to these assumptions and resolving the discrepancies. Accordingly, the aim of the present study is to determine the impact of exercise on the blood pressure of older adults.

**Method:**

In this research, in order to find electronic published papers from 1992 to 2019, the papers published in both domestic and foreign databases including SID, MagIran, IranMedex, IranDox, Gogole Scholar, Cohrane, Embase, Science Direct, Scopus, PubMed, and Web of Science (ISI) were used. Heterogeneity index between the studies was determined based on Cochran test Q(c) and *I*^2^. Considering existence of heterogeneity, random effects model was employed to estimate the standardized subtraction of the mean exercise test score for reduction of blood pressure in the older adults across the intervention group before and after the test.

**Results:**

In this meta-analysis and systematic review, eventually 69 papers met the inclusion criteria. The total number of participants was 2272 in the pre- and postintervention groups when examining the systolic changes and 2252 subjects in the pre- and postintervention groups when inspecting the diastolic changes. The standardized mean difference in examining the systolic changes before the intervention was 137.1 ± 8.09 and 132.98 ± 0.96 after the intervention; when exploring the diastolic changes, the pre- and postintervention values were 80.3 ± 0.85 and 76.0 ± 6.56, respectively, where these differences were statistically significant (*P* < 0.01).

**Conclusion:**

The results of this study indicated that exercise leads to significant reduction in both systolic and diastolic blood pressure. Accordingly, regular exercise can be part of the treatment plan for hypertensive elderly.

## 1. Background

Senescence is a natural course of development in which special physical, psychological, and social changes occur [1]. In other words, senescence refers to spontaneous and irreversible progressive degenerative changes in which both psychological and physical power significantly decline [2]. In the elderly, all organs of the body undergo some degree of degeneration in all of their tasks; for this reason, various chronic diseases occur in the older adults including cardiovascular disease such as hypertension, coronary artery disease, and skeletal diseases such as arthritis, osteoporosis, and cancer [3].

Hypertension is the most common cardiovascular disease in the older adults [4], claiming high healthcare costs [4]. Since pharmacotherapy among the older adults necessitates adhering to various issues, today researchers tend to recommend nonpharmacological methods instead of pharmacotherapy considering the pathological mechanism of hypertension. Nonpharmacological methods include modifying the lifestyle through low sodium diet, low fat diet, increasing potassium as well as calcium intake, weight reduction in obese individuals, daily exercise, and reducing anxiety and fear [5]. Regular exercise at a moderate level for three days per week 30 min/day results in increased longevity, reduced mortality, and reduced development of cardiovascular disease, heart attack, hypertension, arthritis, osteoporosis, depression, and different types of cancer [6]. Regular aerobic exercise leads to reduction of both systolic and diastolic blood pressure by 11 and 8 mmHg. A regular physical activity program should start gradually and sustain for 30–45 min in most days of the week. This level of activity can control hypertension without pharmacotherapy [7].

The impact of aerobic exercises on hypertension has mostly been tested in long-term exercise programs (at least three months) with high intensity and high number of sessions per week (5 days/week). Increase in the number of exercise sessions per week and high intensity of exercise in individuals who are not able to do high intensity activities may be an obstacle to participating in such exercise programs [8]. There are different and sometimes contradictory responses to the numerous questions about the effect of different exercises and their varying intensities on the elderly's blood pressure. Various research studies have reported different results about the impact of exercise on blood pressure considering the type of exercise, its conditions, duration, and frequency within a specific period, and its relationship with blood pressure reduction [9].

In the research by Moraes et al., after three days of aerobic exercise per week for three months in the intervention group, the mean systolic and diastolic blood pressure diminished by 3.2 and 1.2 mmHg, respectively, but no significant change was observed in the mean blood pressure of the control group [10]. In the research by Ferrier et al., the arterial compliance showed resistance against a short aerobic exercise program, and no reduction was found in the blood pressure of patients [11]. The study by Tabara et al. with the aim of comparing aerobic short-term and long-term exercise programs with mild and moderate intensities on cardiovascular indicators of the older adults indicated that the short-term program had no impact on reducing systolic blood pressure, but it decreased the diastolic blood pressure. Long-term program resulted in diminished mean systolic and diastolic blood pressure from 136 to 129 and from 87 to 83, respectively. Also, both the mild and moderate intensity programs were influential for blood pressure reduction [12]. In the research by Westhoff et al., the impact of moderate intensity long-term exercise program was tested on patients with hypertension. The results showed blood pressure decline in the samples, though it was not statistically significant [13].

With regard to the impact of exercise on the blood pressure of the older adults with hypertension, some preliminary studies have been conducted across Asia, Europe, and America, which have found contradictory results. One of the uses of meta-analysis study is to address these assumptions and resolve the contradictions. Although Herrod et al. [14] conducted a meta-analysis study investigating the impact of exercise and other nonpharmacological measures on the blood pressure of the elderly, and this study has not tested the influence of exercise on the blood pressure of the older adults across different continents. Thus, the aim of this study is to determine the impact of exercise on the blood pressure of the older adults with hypertension across meta-analysis.

## 2. Methods

### 2.1. The Methods for Searching Papers

In this investigation, the search was performed on Persian databases including SID, MagIran, IranMedex, and IranDoc along with the international databases of Google Scholar, Cochrane, Embase, Science Direct, Scopus, PubMed, and Web of Science (ISI) with the aim of finding relevant papers without any time constraint (from 1992 to 2019). The list of the references utilized in all papers and the relevant reports found in the previously mentioned electronic search was assessed manually so that other possible references could also be found. The keywords used for searching the references were chosen from The Medical Subject Headings (MeSH) thesaurus. The keywords searched were exercise, resistance training, circuit-based exercise, plyometric exercise, exercise therapy, exercise training, physical activity, and hypertension (both in English and Persian).

((((((((((((Exercise[Title/Abstract]) OR Physical Activity[Title/Abstract]) OR Exercise Training[Title/Abstract]) AND Resistance Training[Title/Abstract]) OR Strength Training[Title/Abstract]) OR Weight-Bearing Exercise Program[Title/Abstract]) AND Circuit-Based Exercise[Title/Abstract]) OR Circuit Training[Title/Abstract]) AND Plyometric Exercise[Title/Abstract]) OR Plyometric Drill[Title/Abstract]) OR Plyometric Training[Title/Abstract]) OR Stretch-Shortening Cycle Exercise [Title/Abstract])))))))))))

### 2.2. The Criteria of Selection of Papers

The papers with the following characteristics were chosen for the meta-analysis: (1) original research papers, (2) clinical trials studies, and (3) availability of full text of papers. For the objectives of this investigation, physical exercise is any bodily activity that enhances or maintains physical fitness and overall health and wellness. It is performed for various reasons including strengthening muscles and the cardiovascular system, honing athletic skills, and weight loss or maintenance, as well as for the purpose of enjoyment [15]. The older adults were defined as individuals above 60 years of age, while hypertensive patients was defined as the patients with a medical diagnosis of hypertension for more than six months (it includes patients with a definite diagnosis of hypertension and does not include patients with prehypertension).

### 2.3. Exclusion Criteria

The selected studies were investigated more accurately. Those conducted as review or those whose sample had not been chosen from the older adults with hypertension or the studies repeated with previous data were removed from the meta-analysis. Eventually, 76 studies entered the third stage, qualitative assessment. Each article was separately reviewed by two reviewers. If the article was rejected by them, they expressed the reason, and if there was any controversy between the reviewers, the article was reviewed by a third referee whose opinion was considered as the final decision. Duplicate publication and multiple publications from the same population were removed using citation management software EndNote (version X7, for Windows, Thomson Reuters).

### 2.4. Qualitative Assessment of the Studies

The quality of the papers was evaluated based on the selected and relevant items of CONSORT checklist, which could be assessed in this study and already mentioned in previous studies (design of study, background and review of literature, place and time of the study, consequence, inclusion criteria, sample size, and statistical analysis). The papers mentioning six to seven criteria were considered of high quality, while those citing two or less of the seven mentioned items were considered as moderate and low quality papers in terms of their methodology [16]. In the present study, 69 papers were included in this systematic review and meta-analysis as being of high and moderate quality, while seven papers which were of low quality were excluded.

### 2.5. Data Extraction

All of the final papers introduced into the meta-analysis process were prepared for extraction by a premade checklist. The checklist included title of paper, name of the first author, year of publication, place of study, sample size of the intervention group, mean systolic and diastolic blood pressure before the intervention, mean systolic and diastolic blood pressure after the intervention, and standard deviation of systolic and diastolic blood pressure both before and after the intervention.

### 2.6. Statistical Analysis

Since the studied index was the impact of exercise on the blood pressure of the elderly, in order to combine the results of different studies, frequency and percentage were used along with standardized mean difference index in every study. In order to investigate homogeneity across studies, *I*^2^ index was used; considering the heterogeneity in the studies, random effects model was used to combine the studies and conduct the meta-analysis. Note that *I*^2^ < 25%, 25–75%, and greater than 75% represent low, medium [16], and high heterogeneity, respectively. *P* < 0.05 was considered as statistically significant. Also, to investigate publication bias, funnel plot and Egger test were used.

## 3. Results

**Figure 1:**
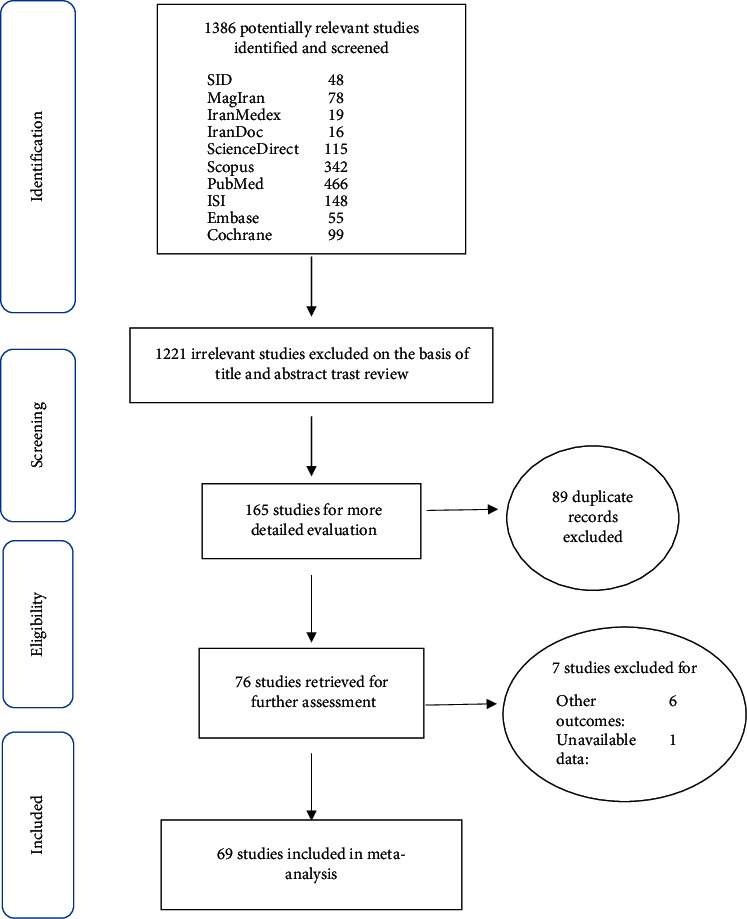
Flow diagram of study selection.

In this study, all studies conducted over the impact of exercise on the blood pressure of older adults were examined systematically without any time constraint and based on the PRISMA instructions. In the preliminary search, 1386 papers were identified; eventually, 69 studies published from 1992 to March 2019 were included in the final analysis (Figure 1).

The total number of participants was 2272 in the pre- and postintervention groups for investigating systolic changes, and 2252, for investigating diastolic changes. The characteristics of the studies included in this systematic review are shown in Table 1.

Based on the available data, for final estimation of the effects of studies, standardized mean difference indices were used in the papers. In the studies that had reported standard deviation ± mean, standardized mean difference index was used in the meta-analysis. The results obtained from meta-analysis showed that across the studies, heterogeneity in investigating systolic changes pre- and postintervention was obtained as *I*^2^ = 98.8 and 98.6, while it was 99.2 and 98.6, respectively, for diastolic changes. Thus, for combination of studies and the final results, random method was used.

**Figure 2:**
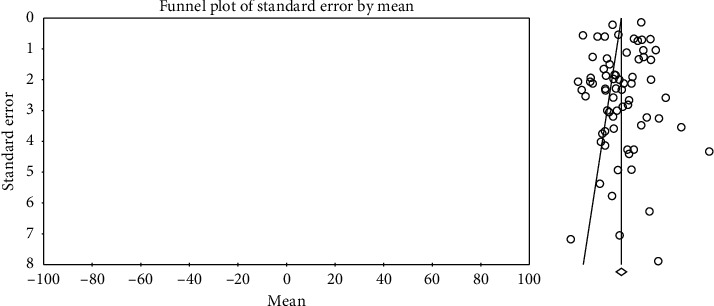
Funnel plot obtained from the studies introduced in the meta-analysis based on the standardized mean difference index for systolic changes preintervention.

**Figure 3:**
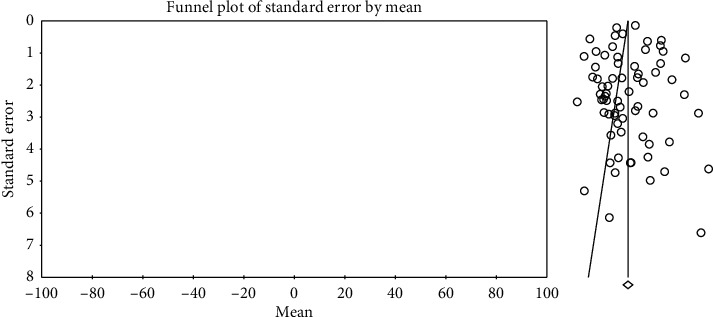
Funnel plot obtained from the studies introduced in the meta-analysis based on the standardized mean difference index for systolic changes postintervention.

**Figure 4:**
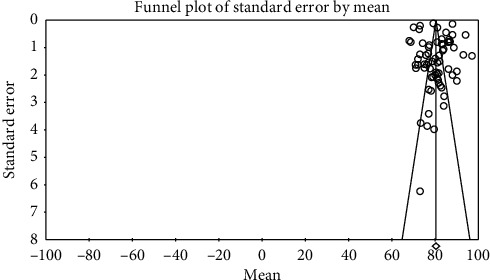
Funnel plot obtained from the studies introduced in the meta-analysis based on the standardized mean difference index for diastolic changes preintervention.

**Figure 5:**
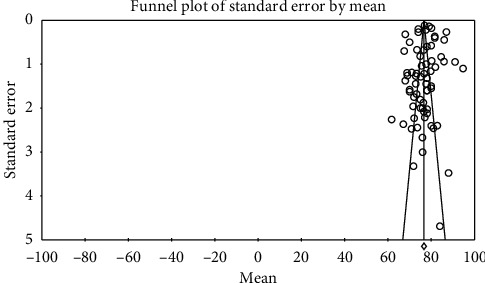
Funnel plot obtained from the studies introduced in the meta-analysis based on the standardized mean difference index for diastolic changes postintervention.

In order to investigate publication bias in the studies, Egger test was used. According to the results of this test, publication bias did not exist in investigating systolic changes pre- (*P*=0.057) and postintervention (*P*=0.713) and investigation of diastolic changes before (*P*=0.943) and after the intervention (*P*=0.522) (Figures 2–5).

**Figure 6:**
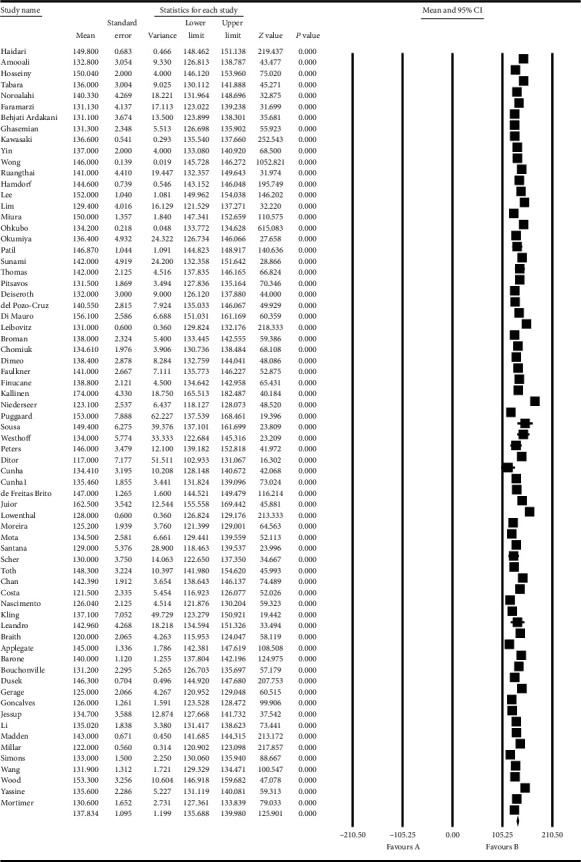
Cumulative diagram obtained from the studies introduced in the meta-analysis based on the standardized mean difference index for systolic changes preintervention.

**Figure 7:**
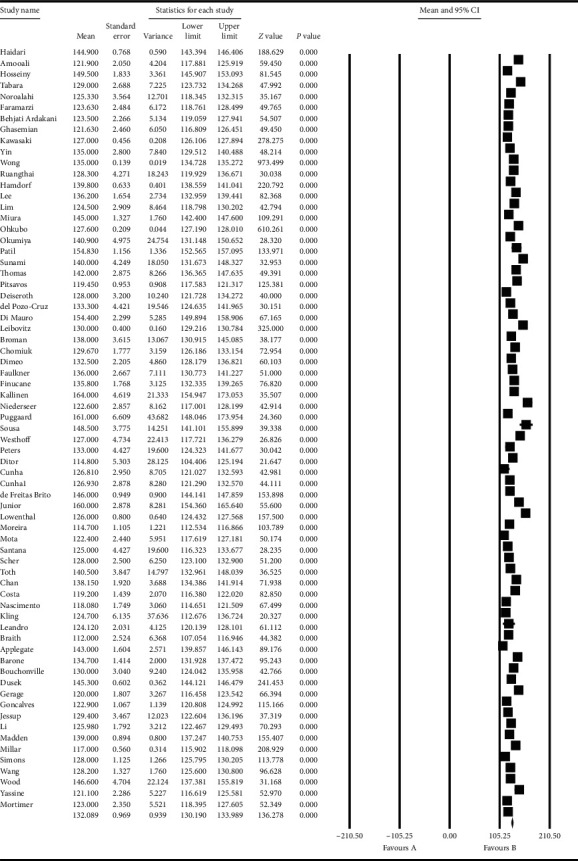
Cumulative diagram obtained from the studies introduced in the meta-analysis based on the standardized mean difference index for systolic changes postintervention.

**Figure 8:**
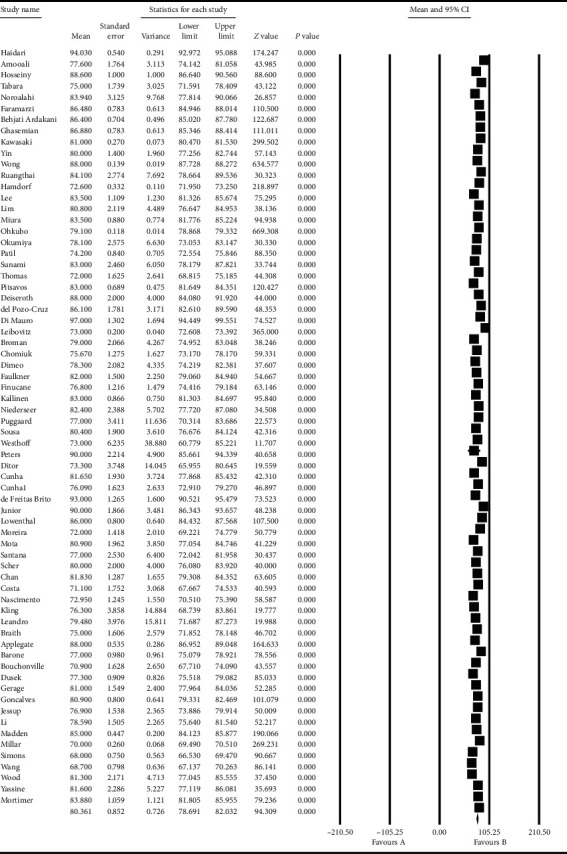
Cumulative diagram obtained from the studies introduced in the meta-analysis based on the standardized mean difference index for diastolic changes preintervention.

**Figure 9:**
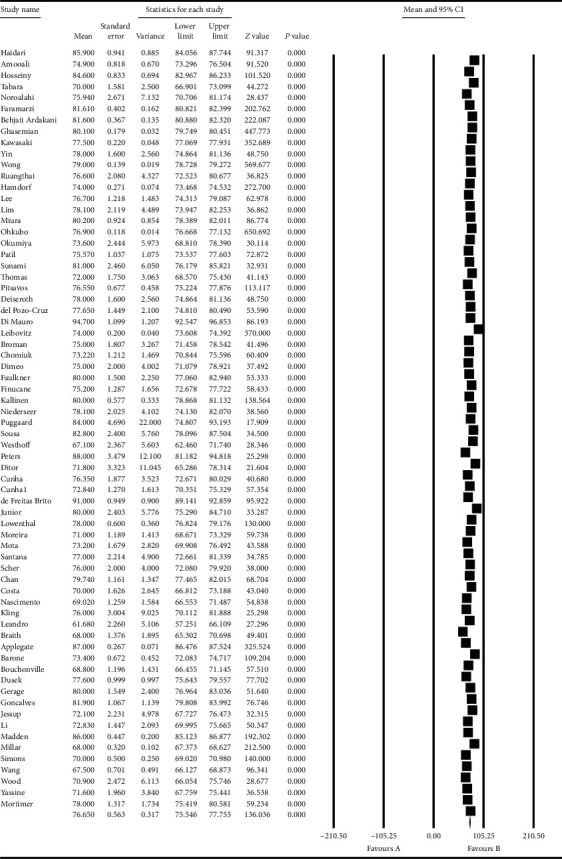
Cumulative diagram obtained from the studies introduced in the meta-analysis based on the standardized mean difference index for diastolic changes postintervention.

Based on the results obtained from the meta-analysis, the standardized mean difference in examining the systolic changes pre- and postintervention was obtained as 137.8 ± 1.09 and 132.08 ± 0.96, while, for diastolic changes, they were 80.3 ± 0.85 and 76.6 ± 0.56, respectively. All of these suggest that exercise leads to diminished hypertension during advanced ages. In the cumulative figures, the standardized mean difference index, confidence interval of 95% in each study, and the final result of the index obtained from combining the studies have been shown. In this diagram, the weight of each study has been shown in the final combined value, where the size of each square is in proportion with the weight that the study has had in the meta-analysis. The horizontal line of each square represents the confidence interval of 95% (Figures 6–9).

**Figure 10:**
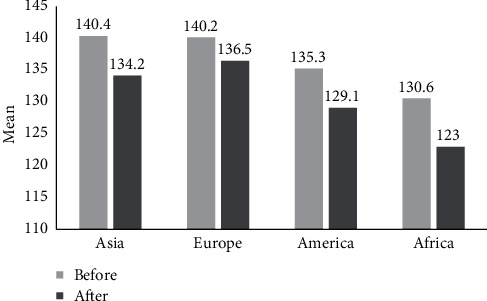
The results of mean systolic blood pressure across different continents for pre-/postintervention.

**Figure 11:**
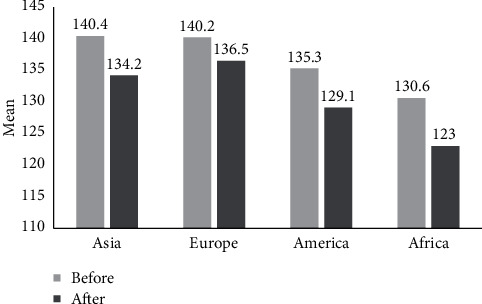
The results of mean diastolic blood pressure across different continents for pre-/postintervention.

According to Table 2 reporting the mean and standard deviation of pre-/postintervention in systolic and diastolic blood pressure changes in terms of different continents (Asia, Europe, Africa, and America), 21, 15, 1, and 32 papers were analyzed in the meta-analysis from Asia, Europe, Africa, and America, respectively. In all of the investigations in terms of the studies carried out in the mentioned continents, exercise led to reduced age-induced hypertension (Figures 10 and 11).

### 3.1. Standard Difference in Mean

**Figure 12:**
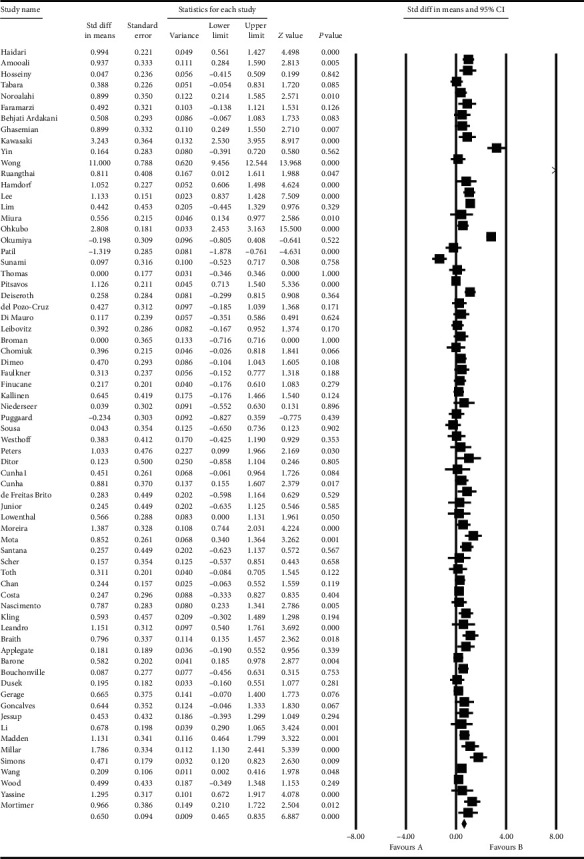
Standard difference in mean between systolic blood pressure changes pre- and postintervention.

**Figure 13:**
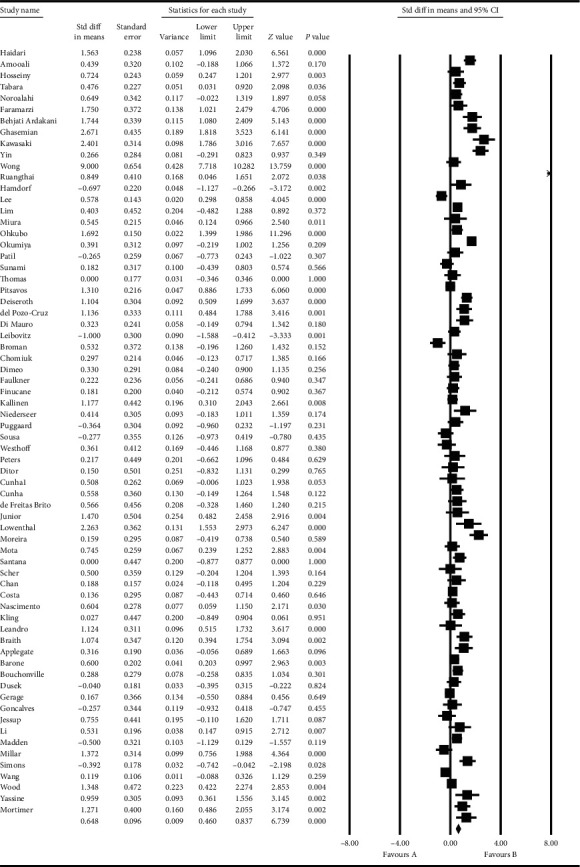
Standard difference in mean between diastolic blood pressure changes pre- and postintervention.

In the study of the mean difference between systolic and diastolic blood pressure changes pre- and postintervention, it was reported that the difference between the systolic blood pressure changes pre- and postintervention was 0.65 ± 0.09, which showed a decrease in the systolic blood pressure after exercise (Figure 12), and the difference between the diastolic blood pressure changes pre- and postintervention was 0.64 ± 0.3609, which showed a decrease in the diastolic blood pressure after exercise (Figure 13).

### 3.2. Subgroup Analysis Based on the Type of Exercise

Subgroup analysis based on the standard difference in mean before and after the intervention according to the type of exercise shows that resistance exercises reduces systolic (0.69 ± 0.1) and diastolic blood pressure (0.73 ± 0.16) more than aerobic exercise (Table 3).

## 4. Discussion

Hypertension is one of the most common diseases in industrial countries [82] and one of the important causes of atherosclerosis, which can cause different problems. In case the treatment is not received, 50% of patients with hypertension die because of coronary artery diseases and congestive heart failure, 33%, because of stroke, and 10–15%, due to renal complications. Further, other organs including the eyes and larger vessels can also be affected [83]. Thus, the aim of the present study is to determine the impact of exercise on the blood pressure of the older adults with hypertension across Asia, Europe, Africa, and America through meta-analysis.

Based on the results obtained from the meta-analysis here, the standardized mean difference in investigating the systolic changes before and after the intervention was 137.8 and 132.08, respectively, and, for diastolic changes, 80.3 and 76.6, respectively. All these suggest that exercise causes a significant decline in age-induced hypertension.

Chronic hypertension adversely affects the myocardial structure and function, inducing a concentric hypertrophy [84]. It seems that the hypertrophic cardiac response to the overpressure is an attempt for normalizing the ventricular walls, thus helping preserve the heart function when undergoing an increased hemodynamic load. This process of hypertrophy is called compensatory hypertrophy [85]. Physical exercise leads to proper adaptation in the cardiovascular system, thereby reducing heart rate, resting heart rate, and increased left ventricle filling, venous return, and stroke volume [86]. In a research by Hinderliter et al., they concluded that after a six-month aerobic exercise program, the left ventricle hypertrophy of patients with hypertension diminished significantly. This reduction of hypertrophy was associated with reduced blood pressure and weight loss of patients. These researchers also found that weight loss is an important factor in mitigating the left ventricle hypertrophy [87]. In addition, Kokkinos et al. observed that aerobic exercise can lead to diminished hypertrophy in hypertensive individuals [88]. Notwithstanding, following aerobic exercise, eccentric contractions occur, whereby the ventricle volume grows; hypertrophy in the ventricle is also possible to occur though to a little extent (in healthy subjects). However, when the subjects are hypertensive, since they have pathologic hypertrophy in the ventricle wall, the mechanism is different. This means that, upon physiological increase in the ventricle dimensions (resulting from aerobic activity) and according to Frank-Starling law, the stroke volume increases and thus pathologic hypertrophy of the ventricular wall diminishes [89].

The present study indicates the results of mean and standard deviation before and after the intervention in changes of systolic and diastolic blood pressure for different continents. These changes have been reported for Asia, Europe, Africa, and America. In all of the investigations conducted across different continents, it was reported that exercise leads to significant reduction of age-induced hypertension. The real mechanism of postactivity hypotension is unknown, and most probably the mechanism is multifactorial. Studies suggest that acute hypotension is mostly associated with diminished peripheral resistance of vessels rather than cardiac output [90]. According to animal and human studies, diminished sympathetic activity occurs after physical exercise [91–93]. Changes in reactivity of vessels are associated with reduced sympathetic conduction for vessel resistance and release of local vasodilator substances (e.g., nitric oxide) in response to muscle contraction and increased blood flow to the muscles. After a heavy physical exercise, reactivity of vessels to alpha-adrenergic stimulation diminishes [94]. The local release of nitric oxide, prostaglandins, and adenosine increases during physical activity, thus facilitating peripheral postactivity vasodilation [95].

Postexercise hypertension is a result of physical activity, and daily changes of blood pressure do not affect its reduction. The density and volume of physical exercise play an important role in hemodynamic and thermal regulation as well as regulation of neurological reactions of the body during activity [96]. Also, Syme et al. reported that the intensity of physical activity influences the duration of hypotension and has a direct relationship with it [97]. Studies show that possibly factors such as diminished plasma volume, increased vasodilation substances, changes in the hormones affecting blood pressure including vasopressin, angiotensin 2 and renin, and peripheral vasodilation resulting from elevated central temperature are effective in inducing hypotension [98].

Use of physical exercise in the long run functions as a no pharmacological method for blood pressure reduction at rest or during daily physical activities [99]. In the elderly, exercise may be a more suitable method for controlling blood pressure because of low cost and not interfering with other treatments. Through exercise and physical activity, the adverse physiological effects that occur with the aging can be mitigated and the quality of life can be improved [100]. One session of mild or moderate intensity exercise can lead to blood pressure fall after exercise in hypertensive individuals, which is called postexercise hypotension [101].

In the human, postexercise hypotension is observed in response to several types of exercise in which large muscles are active including jogging, cycling, running, and swimming [102].

Through exercise, the oxidation capacity of muscles increases, whereby the aerobic biochemical system is stimulated to create adaptation. All these result in enhanced oxygen uptake in the body. Some diseases cause inhibition of oxygen in any of the above stages and reduce the functional capacity. However, aerobic exercises are able to create physiological adaptation in the efficiency of the aerobic energy system. They also enhance the functional ability of the person and improve the functional capacity even under progression conditions of the disease. Other advantages of regular exercise in this group of patients include increased power, improved body posture, diminished fatigue, improved mood, increased self-confidence, and sense of well-being. Doing physical exercise increases the persons' independence thereby leading to improved quality of life [103, 104].

Since these methods are easy to learn and possible to perform for almost all patients and there is no need to special equipment or cost, and even the patient can perform them in a lying position, patients can learn these methods easily and do them at home. By benefiting from the impacts of exercise methods such as greater blood perfusion to the muscles and reduction of stress and anxiety, their hypertension would diminish and their performance would be boosted. Also, through training patients on these methods and supervision on the way they should be practiced, the disturbing symptoms of this disease can be mitigated. Thus, by educating this method to both the healthcare team and patients, effective steps can be taken to alleviate this disorder.

It is recommended that relevant specialists benefit from regular aerobic exercise as a complementary treatment alongside pharmacotherapy to help patients with hypertension.

One of the limitations of the present research was completing the sheet of doing the exercise at home by the patients in the papers introduced to this meta-analysis, which may have been affected by the psychological status or inadequate care of the samples. Nevertheless, in some papers, checking whether exercise was done was followed up through a weekly in-person meeting with the patients.

## 5. Conclusion

The results of this study indicated that exercise significantly reduces blood pressure in the older adults across different continents. Accordingly, regular physical exercise can be part of the healthcare program of older adults with hypertension.

## Figures and Tables

**Table 1:** Specifications of studies entered into the meta-analysis.

Author, year, and reference	Place of study	Sample size	Mean ± SD of before SBP	Mean ± SD of after SBP	Mean ± SD of before DBP	Mean ± SD of after DBP	Quality
Haidari, 2014, [17]	Iran	46	149.8 ± 4.63	144.9 ± 5.21	94.03 ± 3.66	85.9 ± 6.38	High
Amooali, 2015, [18]	Iran	20	132.8 ± 13.66	121.9 ± 9.17	77.6 ± 7.89	74.9 ± 3.66	High
Hosseiny, 2007, [8]	Iran	36	150.04 ± 12	149.5 ± 11	88.6 ± 6	84.6 ± 5	High
Tabara, 2007, [12]	Japan	40	136 ± 19	129 ± 17	75 ± 11	70 ± 10	High
Noroalahi, 2019, [19]	Iran	18	140.33 ± 18.1	125.33 ± 15.1	83.94 ± 13.26	75.94 ± 11.33	High
Faramarzi, 2012, [20]	Iran	20	131.13 ± 18.5	123.63 ± 11.1	86.48 ± 3.5	81.61 ± 1.8	High
Behjati Ardakani, 2018, [21]	Iran	24	131.1 ± 18	123.5 ± 11.1	86.4 ± 3.45	81.6 ± 1.8	High
Ghasemian, 2013, [22]	Iran	20	131.3 ± 10.5	121.63 ± 11	86.88 ± 3.5	80.1 ± 0.8	High
Kawasaki, 2011, [23]	Japan	35	136.6 ± 3.2	127 ± 2.7	81 ± 1.6	77.5 ± 1.3	Medium
Yin, 1998, [24]	Japan	25	137.0 ± 10	135 ± 14	80 ± 7	78 ± 8	Medium
Wong, 2019, [25]	Korea	52	146 ± 8.1	135 ± 1	88 ± 1	79 ± 1	High
Ruangthai, 2019, [26]	Thailand	13	141 ± 15.9	128.3 ± 15.4	84.1 ± 10	76.6 ± 7.5	High
Hamdorf, 1999, [27]	Australia	—	144.6 ± 4.9	139.8 ± 4.2	72.6 ± 2.2	74 ± 1.8	Medium
Lee, 2007, [28]	Taiwan	102	152 ± 10.5	136.2 ± 16.7	83.5 ± 11.2	76.7 ± 12.3	High
Lim, 2015, [29]	Korea	10	129.4 ± 12.7	124.5 ± 9.2	80.8 ± 6.7	78.1 ± 6.7	High
Miura, 2015, [30]	Japan	45	150 ± 9.1	145 ± 8.9	83.5 ± 5.9	80.2 ± 6.2	High
Ohkubo, 2001, [31]	Japan	121	134.2 ± 2.4	127.6 ± 2.3	79.1 ± 1.3	76.9 ± 1.3	High
Okumiya, 1996, [32]	Japan	21	136.4 ± 22.6	140.9 ± 22.8	78.1 ± 11.8	73.6 ± 11.2	High
Patil, 2015, [33]	India	30	146.87 ± 5.72	154.83 ± 6.33	74.2 ± 4.6	75.57 ± 5.68	High
Sunami, 1999, [34]	Japan	20	142 ± 22	140 ± 19	83 ± 11	81 ± 11	Medium
Thomas, 2005, [35]	Hong Kong	64	142 ± 17	142 ± 23	72 ± 13	72 ± 14	Medium
Pitsavos, 2011, [36]	Greece	52	131.5 ± 13.48	119.45 ± 6.87	83 ± 4.97	76.55 ± 4.88	High
Deiseroth, 2019, [37]	Switzerland	25	132 ± 15	128 ± 16	88 ± 10	78 ± 8	High
del Pozo-Cruz, 2012, [38]	España	21	140.55 ± 12.9	133.3 ± 20.26	86.1 ± 8.16	77.65 ± 6.64	High
Di Mauro, 1998, [39]	Italy	35	156.1 ± 15.3	154.4 ± 13.6	97 ± 7.7	94.7 ± 6.5	Medium
Leibovitz, 2005, [40]	Israel	25	131 ± 3	130 ± 2	73 ± 1	74 ± 1	High
Broman, 2006, [41]	Sweden	15	138 ± 9	138 ± 14	79 ± 8	75 ± 7	High
Chomiuk, 2013, [42]	Poland	44	134.61 ± 13.1	129.67 ± 11.7	75.67 ± 8.46	73.22 ± 8.04	High
Dimeo, 2012, [43]	Germany	24	138.4 ± 14.1	132.5 ± 10.8	78.3 ± 10.2	75 ± 9.8	High
Faulkner, 2013, [44]	New Zealand	36	141 ± 16	136 ± 16	82 ± 9	80 ± 9	Medium
Finucane, 2010, [45]	UK	50	138.8 ± 15	135.8 ± 12.5	76.8 ± 8.6	75.2 ± 9.1	High
Kallinen, 2002, [46]	Finland	12	174 ± 15	164 ± 16	83 ± 3	80 ± 2	Medium
Niederseer, 2011, [47]	Austria	22	123.1 ± 11.9	122.6 ± 13.4	82.4 ± 11.2	78.1 ± 9.5	High
Puggaard, 2000, [48]	Denmark	22	153 ± 37	161 ± 31	77 ± 16	84 ± 22	High
Sousa, 2013, [49]	Portugal	16	149.4 ± 25.1	148.5 ± 15.1	80.4 ± 7.6	82.8 ± 9.6	High
Westhoff, 2008, [13]	Germany	12	134 ± 20	127 ± 16.4	73 ± 21.6	67.1 ± 8.2	High
Peters, 2006, [50]	USA	10	146 ± 11	133 ± 14	90 ± 7	88 ± 11	High
Ditor, 2005, [51]	Canada	8	117 ± 20.3	114.8 ± 15	73.3 ± 10.6	71.8 ± 9.4	High
Cunha, 2011, [52]	Brazil	30	134.41 ± 17.5	126.81 ± 16.1	81.65 ± 10.57	76.35 ± 10.28	High
Cunha, 2012, [53]	Brazil	16	135.46 ± 7.42	126.93 ± 11.5	76.09 ± 6.49	72.84 ± 5.08	High
de Freitas Brito, 2014, [54]	Brazil	10	147 ± 4	146 ± 3	93 ± 4	91 ± 3	Medium
Júnior, 2019, [55]	Brazil	10	162.5 ± 11.2	160 ± 9.1	90 ± 5.9	80 ± 7.6	High
Lowenthal, 2004, [56]	USA	25	128 ± 3	126 ± 4	86 ± 4	78 ± 3	High
Moreira, 2016, [57]	Brazil	23	125.2 ± 9.3	114.7 ± 5.3	72 ± 6.8	71 ± 5.7	High
Mota, 2013, [58]	Brazil	32	134.5 ± 14.6	122.4 ± 13.8	80.9 ± 11.1	73.2 ± 9.5	High
Santana, 2011, [59]	Brazil	10	129 ± 17	125 ± 14	77.0 ± 8	77 ± 7	High
Scher, 2010, [60]	Brazil	16	130 ± 15	128 ± 10	80 ± 8	76 ± 8	High
Toth, 2006, [5]	USA	50	148.3 ± 22.8	140.5 ± 27.2	—	—	Medium
Chan, 2018, [61]	USA	82	142.39 ± 17.3	138.15 ± 17.3	81.83 ± 11.65	79.74 ± 10.51	High
Costa, 2019, [62]	Brazil	23	121.5 ± 11.2	119.2 ± 6.9	71.1 ± 8.4	70 ± 7.8	High
Nascimento, 2019, [63]	Brazil	27	126.04 ± 11.04	118.08 ± 9.09	72.95 ± 6.47	69.02 ± 6.54	High
Kling, 2019, [64]	USA	10	137.1 ± 22.3	124.7 ± 19.4	76.3 ± 12.2	76 ± 9.5	High
Leandro, 2019, [65]	Brazil	24	142.96 ± 20.91	124.12 ± 9.95	79.48 ± 19.48	61.68 ± 11.07	High
Braith, 1994, [66]	USA	19	120 ± 9	112 ± 11	75 ± 7	68 ± 6	Medium
Applegate, 1992, [67]	USA	56	145 ± 10	143 ± 12	88 ± 4	87 ± 2	Medium
Barone, 2009, [68]	USA	51	140 ± 8	134.7 ± 10.1	77 ± 7	73.4 ± 4.8	High
Bouchonville, 2014, [69]	USA	26	131.2 ± 11.7	130 ± 15.5	70.9 ± 8.3	68.8 ± 6.1	High
Dusek, 2008, [70]	USA	61	146.3 ± 5.5	145.3 ± 4.7	77.3 ± 7.1	77.6 ± 7.8	High
Gerage, 2013, [71]	Brazil	15	125 ± 8	120 ± 7	81 ± 6	80 ± 6	Medium
Goncalves, 2014, [72]	Brazil	17	126 ± 5.2	122.9 ± 4.4	80.9 ± 3.3	81.9 ± 4.4	High
Jessup, 1998, [73]	USA	11	134.7 ± 11.9	129.4 ± 11.5	76.9 ± 5.1	72.1 ± 7.4	High
Li, 2005, [74]	USA	54	135.02 ± 13.51	125.98 ± 13.1	78.59 ± 11.06	72.83 ± 10.63	Medium
Madden, 2010, [75]	Canada	20	143 ± 3	139 ± 4	85 ± 2	86 ± 2	High
Millar, 2008, [76]	Canada	25	122 ± 2.8	117 ± 2.8	70 ± 1.3	68 ± 1.6	High
Simons, 2006, [77]	USA	64	133 ± 12	128 ± 9	68 ± 6	70 ± 4	Medium
Wang, 2011, [78]	USA	180	131.9 ± 17.6	128.2 ± 17.8	68.7 ± 10.7	67.5 ± 9.4	High
Wood, 2001, [79]	USA	11	153.3 ± 10.8	146.6 ± 15.6	81.3 ± 7.2	70.9 ± 8.2	High
Yassine, 2009, [80]	USA	24	135.6 ± 11.2	121.1 ± 11.2	81.6 ± 11.2	71.6 ± 9.6	High
Mortimer, 2011, [81]	South Africa	15	130.6 ± 6.4	123 ± 9.1	83.88 ± 4.1	78 ± 5.1	High

**Table 2:** The mean and standard deviation of pre-/postintervention in systolic and diastolic blood pressure changes across different continents.

Blood pressure changes	Continents	Number of articles	*I* ^2^	Sample size	Egger test	Mean ± SD
Systole (before intervention)	Asia	21	99.2	806	0.706	140.4 ± 1.7
	Europe	15	94.1	411	0.051	140.2 ± 2.4
	America	32	98	1040	0.503	135.3 ± 1.8
	Africa	1	0	15	—	130.6 ± 1.6

Systole (after intervention)	Asia	21	99	806	0.627	134.2 ± 1.8
	Europe	15	96.1	411	0.119	136.5 ± 2.4
	America	32	98.6	1040	0.793	129.1 ± 2.1
	Africa	1	0	15	—	123 ± 2.3

Diastole (before intervention)	Asia	21	99.5	806	0.990	81.8 ± 1.3
	Europe	15	98	411	0.051	81.2 ± 2.01
	America	31	98.6	990	0.138	78.7 ± 1.5
	Africa	1	0	15	—	83.8 ± 1.05

Diastole (after intervention)	Asia	21	97.7	806	0.797	78.1 ± 0.56
	Europe	15	97	411	0.111	77.9 ± 1.4
	America	31	99.1	990	0.207	75.06 ± 1.6
	Africa	1	0	15	—	78 ± 1.3

**Table 3:** Subgroup analysis based on type of exercise.

Type of exercise		Number of articles	Sample size	*I* ^2^	Egger test	Std. difference in mean (before and after intervention)
Aerobic exercise	Systolic blood pressure	48	1734	94.4	0.715	0.69 ± 0.16
	Diastolic blood pressure			89.5	0.178	0.64 ± 0.11

Resistance exercises	Systolic blood pressure	20	494	54.6	0.051	0.69 ± 0.1
	Diastolic blood pressure			82.8	0.053	0.73 ± 0.16

## Data Availability

Datasets are available from the corresponding author upon reasonable request.

## Abbreviations

SID: Scientific information database
CONSORT: Consolidated standards of reporting trials
PRISMA: Preferred Reporting Items for Systematic Reviews and Meta-analysis.
